# Prognostic Value of Serum Copper for Post-Stroke Clinical Recovery: A Pilot Study

**DOI:** 10.3389/fneur.2018.00333

**Published:** 2018-05-30

**Authors:** Rosanna Squitti, Mariacristina Siotto, Giovanni Assenza, Nadia M. Giannantoni, Mauro Rongioletti, Filippo Zappasodi, Franca Tecchio

**Affiliations:** ^1^Molecular Markers Laboratory, IRCCS Istituto Centro San Giovanni di Dio Fatebenefratelli, Brescia, Italy; ^2^IRCCS Fondazione Don Carlo Gnocchi, Milan, Italy; ^3^Clinical Neurology, Campus Biomedico University of Rome, Rome, Italy; ^4^Neurocenter of Southern Switzerland, Civic Hospital, Lugano, Switzerland; ^5^Laboratory of Electrophysiology for Translational neuroScience (LET’S), ISTC–CNR, Rome, Italy; ^6^Department of Biology Medicine, Research and Development Division, Fatebenefratelli Hospital, Isola Tiberina, Rome, Italy; ^7^Department of Neuroscience, Imaging and Clinical Sciences, “G. d’Annunzio” University of Chieti-Pescara, Chieti, Italy; ^8^Institute for Advanced Biomedical Technologies, “G. d’Annunzio” University of Chieti-Pescara, Chieti, Italy; ^9^Institute of Neurology, Catholic University of the Sacred Heart, Rome, Italy

**Keywords:** ischemic stroke, functional recovery, serum markers, oxidative stress, metal metabolism

## Abstract

The clinical course after ischemic stroke can vary considerably despite similar lesions and clinical status at the onset of symptoms, suggesting that individual factors modulate clinical recovery. Here, we sought to test the working hypothesis that elevated copper values provide prognostic information, and specifically predict worse clinical recovery. We further sought to support previous findings regarding metal metabolism in acute stroke. We assessed total antioxidant status, oxidative stress factors (peroxides) and metal metabolism markers (iron, copper, ceruloplasmin concentration and activity, ferritin, and transferrin) in the acute phase (2–10 days from symptom onset) in 30 patients affected by unilateral middle cerebral artery (MCA) stroke. A longitudinal assessment of clinical deficit was performed in the acute and stabilized phases (typically 6 months post-stroke) using the National Institutes of Health Stroke Scale (NIHSS). In identifying recovery-related factors, we considered effective recovery (ER), calculated as the ratio between actual NIHSS recovery and the total potential recovery. This allows an estimation of the actual recovery adjusted for the patient’s initial condition. In the acute phase, clinical severity was correlated with increased peroxide concentrations, and lower iron levels. Less successful clinical recovery was correlated with increased acute copper levels, which entered a multiple regression model that explained 24% of ER variance. These pilot data suggest that, in the acute phase of an ischemic stroke, copper may provide useful information about clinical recovery.

## Introduction

Ischemic stroke is the rapid loss of brain function due to a reduction in the blood supply to the brain. It is among the prevalent causes of mortality and disability in adults and elderly people (1). In the weeks and months after the onset of symptoms, the clinical course of stroke patients can vary largely even in patients with similar clinical pictures and lesion characteristics (2). For this reason, exploring diverse biomarkers with recovery prognostic value can provide information about potential interventions, which can be adapted to personalize acute and subacute treatments.

Brain blood flow interruption during ischemic insult triggers multiple inflammatory, oxidative stress, and immune responses, for which trace metals and antioxidant systems are required. Free radicals production, disturbed metal homeostasis and glutamatergic excitotoxicity are among the processes activated during the reperfusion phase, with complex relationships with the vital mechanisms limiting the damage within the penumbra, the neighboring area to the ischemic core. Excessive concentrations of divalent metal ions, such as zinc, copper, iron along with the cation calcium, are known mediators of damage in acute ischemic stroke (3–5), through excitotoxicity leading to neuronal death (6, 7). Iron homeostasis is pivotal in maintaining normal brain functions because of the high oxygen requirement of this organ, which further increases during ischemic stroke, placing higher demands on iron transport and utilization within the preserved regions of the brain (8, 9). However, excess metals in the blood and their movement from the blood to the brain can concur with brain insult, when the blood–brain barrier (BBB) is damaged. Iron and transferrin (Tf) withdrawal in serum during the acute phase after ischemic stroke have been reported (10). Recent literature reports that more severe clinical states correlated with increased peroxide concentrations and lower iron levels (11, 12). Moreover, local acidosis may facilitate further metal release from the transporting proteins. In these conditions, iron and copper can produce neuronal injury by catalyzing the conversion of superoxide and hydrogen peroxide into highly reactive radicals. Three previous studies discovered significantly elevated contents of both total copper and “free” copper (generally understood as copper not bound to ceruloplasmin, nCp-Cu) in the serum of cerebral ischemic stroke patients (10, 13, 14), while others described the direct role of copper in learning and memory tied to plasticity mechanisms largely mediated by glutamate neurotransmission (7, 15–17). In both healthy people and Alzheimer’s disease patients, we observed a link between circulating copper and glutamate-mediated neural transmission (18, 19). Besides the phenomena impacting the clinical severity in the acute phase, largely uncoupled mechanisms sustain clinical recovery (20–22), also mediated by brain plasticity phenomena (23, 24). However, no study has yet explored serum early metal and oxidative stress biomarkers in relation to post-stroke clinical recovery. Here, we present a prospective study of a panel of metal and oxidative stress biomarkers in a group of patients after acute ischemic stroke. Our working hypothesis is that elevated copper values can provide prognostic information about a less successful clinical recovery. To resolve this question, we designed a prolonged 6-month clinical follow-up study. Our secondary aim was to confirm previous results about iron, copper, ceruloplasmin, ferritin, and Tf trends in the ischemic acute phase.

## Materials and Methods

### Subjects

We enrolled 30 patients, who were admitted to hospitals in Rome because of a first-ever mono-hemispheric ischemic stroke affecting the middle cerebral artery (MCA) territory (Table 1). The inclusion criteria were as follows: clinical evidence of sensory-motor deficit of the upper limb and neuroradiological diagnosis of ischemic brain damage in MCA territory. The exclusion criteria were as follows: previous stroke in their clinical history; neuroradiological evidence of involvement of both hemispheres, and brain hemorrhage; dementia or aphasia severe enough to impair patients’ compliance with the procedures; and acute infectious processes, like pneumonia or urinary tract infection.

**Table 1:** Patients’ personal and clinical data (*n* = 30).

Age: mean (SD), years	70.9 (10.7)
Sex (%)	70% male, 30% female
**Neuroradiological picture**	
Lesion site (hemisphere)	60% left, 40% right
Lesion class: Cortical	10%
Subcortical	30%
Cortico–subcortical	60%
**Clinical picture**	
NIHSS at t0: median (5°, 95° percentile), score	5 (1, 22)
NIHSS at t1: median (5°, 95° percentile), score	2 (0, 18)
Effective recovery: median (5°, 95° percentile) %	63 (0, 100)
**Vascular risk factors (%)**	
Smoking	18%
Diabetes	8%
Hypertension	70%
Cardiopathy (atrial fibrillation)	33% (13% AF)
Hyperlipidemia	56%
Familiarity	5%

*t0 is the time of acute phase analyses (2–10 days); t1 is the time of stabilized phase clinical examination (about 6 months). NIHSS, National Institute for Health Stroke Scale scores*.

Patients received the best clinical care according to the Italian stroke guidelines (SPREAD). None of them received thrombolysis because they were admitted after the time limit from the onset of symptoms to allow thrombolysis.

The ethics committees of our hospitals approved the experimental protocol and all subjects signed a written informed consent form before participating in accordance with the Declaration of Helsinki.

### Study Design and Sample Size Estimate

Our primary aim was to investigate whether acute phase serum copper could provide prognostic information for clinical recovery.

In determining how to assess clinical recovery, we took into account that after symptoms onset, stroke patients’ clinical course can vary largely even in patients with similar clinical pictures and lesion characteristics (2). The phenomena impacting the clinical severity in the acute phase were observed largely uncoupled from those that sustain the clinical recovery (20–22). The observation that phenomena impacting the clinical severity in the acute phase are largely uncoupled from those that sustain the clinical recovery (20–22) supports our efforts in identifying “prognostic markers of recovery,” as independently as possible from the acute phase clinical state. In fact, we adhere to the idea that, given the clinical state in the acute phase as a non-modifiable condition, the features of the acute condition can be exploited more actively to identify factors that can guide better early interventions, intended to raise the patients’ recovery chances toward higher clinical improvements. Overall, we search here for acute phase metal and oxidative stress biomarkers, which—starting from the non-modifiable early clinical state—assess patients’ individual recovery ability. Along this line of reasoning, we have assessed the clinical recovery *via* a “normalized” marker of “effectiveness” called effective recovery, ER (25, 26), that aims to be, to the highest degree possible, “independent of” the acute phase clinical state. The National Institutes of Health Stroke Scale (NIHSS) clinical scores were thus collected in post-acute stabilized phase after about 6 months (t1, 5.2 ± 1.9 months). ER was calculated as the percentage of actual improvement compared to the total possible improvement, taking into account that NIHSS = 0 corresponds to the absence of clinical symptoms:

ER=100∗(NIHSS at t0−NIHSS at t1)/(NIHSS at t0−0).

To assess our working hypothesis, we enrolled 30 cases as an acceptable sample. If the true association between ER and baseline copper were large (*r* = 0.5, indicating that 25% of ER variance could be explained by copper), a sample of 30 cases allows us to reach a power of 0.86, which would identify the results as significant (with two-sided alpha set at 0.05). This also means that our sample size is not able to recognize significant lower associations.

### Data Collection

Patients’ National Institutes of Health Stroke Scale (NIHSS) scores, brain magnetic resonance imaging (MRI) and blood samples were collected on the same day, after the patients’ vital parameters reached stabilization and before day 10 after symptoms onset (t0, 4.2 ± 3.7 days).

The brain MRI was carried out at 1.5 T by Spin-Echo, Turbo Spin-Echo, and Fluid-Attenuated Inversion Recovery sequences, collected contiguously with 5-mm thick slices on the sagittal, coronal, and axial planes. The identification of the lesion site was performed on axial slices. If the cortical gray matter was involved and all subcortical structures were spared, the lesions were classified as “cortical” (C); when white matter, internal capsule, thalamus, or basal ganglia were affected, the classification was “subcortical” (S); and when both the cortical and subcortical structures were involved, the lesion class was “cortico–subcortical” (CS) (Table 1).

We described the results in a complete way by considering both acute and stabilized clinical conditions in relationship with biochemical factors, as well as presenting their individual values. Nevertheless, we focused the prognostic analysis on ER, in an attempt to disentangle “recovery” from the absolute acute clinical state in assessing the prognostic value of acute phase copper.

### Biochemical Investigation

After vital parameter stabilization, between 2 and 10 days from the onset of stroke symptoms (t0), the blood samples were collected in the early morning (7:30–9:00 a.m.) after an overnight fast to also standardize the assessment of those biochemical variables that are affected by the circadian cycle and food intake. Sera samples were separated by centrifugation (3,000 rpm, 10 min, and 4°C). They were then divided into 0.5 mL aliquots and rapidly stored at −80°C. Subjects’ samples and reference samples were thawed just before the assay.

All the analyses of serum were performed in duplicate using the multiple biochemical analyzer Horiba Pentra 400 (ABX Diagnostic, Montpellier, France). Concentration of immunological ceruloplasmin (iCp) was measured with an immunoturbidimetric assay (Futura System SRL, Rome, Italy) and was calibrated against the international reference preparation (ERM 470) (27). Tf levels were measured by immunoturbidimetric assay (28) and ferritin was measured by latex-enhanced turbidimetric immunoassay (29) (Horiba ABX, Montpellier, France). In these assays, serum was mixed with a purified immunoglobulin fraction rabbit anti-human Cp antiserum or rabbit anti-human Tf antibody solution. The resulting immune complexes were measured on the base of variation in turbidimetry. Iron was measured by photometric test using Ferene (Horiba ABX, Montpellier, France) (30). Serum copper concentration was measured with the colorimetric assay of Abe et al. (Randox Laboratories, Crumlin, UK) (31). These results were confirmed by atomic absorption spectrophotometry measurements of copper in serum samples (A Analyst 600, Perkin-Elmer, Norwalk, CT, USA, equipped with graphite furnace). The enzymatic activity of Cp (eCp) was measured following an automation of the manual Schosinsky o-dianisidine eCp assay (32, 33) adapted from our laboratory for multiple biochemical analyzers and described in detail elsewhere (34). Total antioxidant blood capacity (TAS) was assayed by the TAS kit (Randox Laboratories, Crumlin, UK), based on published methods (35). Hydro-peroxide content was assessed using a d-ROMs test (Diacron, Italy) and expressed in arbitrary units (U.CARR), 1 U.CARR corresponding to 0.08 mg/100 mL of hydrogen peroxide [see Ref. (10)].

### Statistical Analysis

As a preliminary step, we compared the results we gained in a previous study of ours on metal metabolism in the acute stroke phase (10) with those obtained in this new patients’ sample. Thus, we calculated the correlations of total antioxidant status, oxidative stress factors, and metal markers with NIHSS at t0, by Spearman’s correlation, since the distribution of the clinical scale does not fit a Gaussian distribution. To complete the check, we considered also nCp-Cu, calculated from the equation provided by Walshe [appendix of Ref. (36)], based on the measures of concentration of total copper and iCp in serum.

Furthermore, to complete the description of the processes in the stabilized phases, we showed the correlations of the biochemical variables with NIHSS in t1 and ER. The effects of sex and age were not taken into account as a result of the small size of the sample under study.

To give a picture of the variables under study in our patients, data were expressed as mean and SD or, if they were not normally distributed, as median and interquartile range (IQR: 25–75th percentiles). For each biochemical variable, we considered the normal reference range, as reported in biochemical assays.

Statistical analyses were performed using SPSS v 16 statistical software (Chicago, IL, USA).

## Results

### Clinical Status

Clinical severity in the acute phase was widely variable in the studied population (minimum 1, maximum 22) and the recovery abilities ranged from 0 to 100% (Table 1). As expected, NIHSS at t0 correlated with NIHSS at t1 (Spearman’s rho = 0.729, *p* < 0.001). In accordance with the normalization introduced in the ER definition to study the recovery abilities “independently of” the acute phase clinical state, the NIHSS at t0 did not display a significant correlation with the ER (rho = −0.244, *p* = 0.194). On the contrary, NIHSS in stabilized phase (NIHSS1) correlated with ER (rho = 0.777, *p* < 0.001), indicating that worse clinical severities corresponded to lower levels of recovery.

### Biochemical Variables Profile in the Acute Phase

Serum peroxides were higher than the relative upper limit of normal (Table 2).

**Table 2:** Values of the biological variables in the stroke acute phase (t0).

	Mean	SD	Reference values
Copper (μmol/L)	15.6	4.76	11–24.4
Transferrin (g/L)	2.5	0.38	2.0–3.6
Ceruloplasmin, immunological ceruloplasmin (mg/dL)	29.2	5.9	22–61
Copper not bound to ceruloplasmin, nCp-Cu (μmol/L)	**1.8**	3.7	<1.6^§^
Iron (μg/dL)	69.6	53.2	F:39–149; M: 40–120
Peroxides, dRoMs (UCARR)	**377.8**	109.5	250–300
Total antioxidant status, TAS (mmol/L)	1.3	0.18	1.3–1.77
Ferritin (ng/mL)	173.4	117.75	F:10–120; M: 20–250

*^§^Reference values are reported as in biochemical assays*.

*Bold represents value out of normal reference range*.

Iron and peroxide levels correlated with the clinical severity in the acute phase (NIHSS0, Table 3). Lower iron levels corresponded to worse clinical conditions. Conversely, the higher the peroxide, the higher the clinical severity.

**Table 3:** Correlations between clinical and biological variables.

	NIHSS0	National Institutes of Health Stroke Scale (NIHSS1)	Copper
Copper (μmol/L)	0.182	**0.416**	
	*0.336*	*0.022*	

Transferrin (g/L)	−0.292	−0.269	−0.245
	*0.124*	*0.159*	*0.199*

Ceruloplasmin (mg/dL)	0.330	0.342	0.612*
	*0.075*	*0.064*	<*0.001*

Copper not bound to ceruloplasmin, nCp-Cu (μmol/L)	−0.056	0.263	0.811*
	*0.768*	*0.160*	<*0.001*

Iron (μg/dL)	−**0.408**	−0.256	−0.224
	*0.028*	*0.180*	*0.244*

Peroxides (UCARR)	0.531*	**0.416**	**0.450**
	*0.003*	*0.022*	*0.013*

Total antioxidant capacity (mmol/L)	−0.362	−0.098	0.036
	*0.058*	*0.620*	*0.855*

Ferritin (ng/mL)	−0.073	0.098	0.030
	*0.753*	*0.672*	*0.897*

*Correlations between biological variables of metal status evaluated in the stroke acute phase (t0) and the clinical assessment at t0 (NIHSS0) and in the stabilized phase (NIHSS1) by non-parametric tests (Spearman’s rho, p)*.

*In bold correlations with significance below p = 0.05, italics font expresses the significance of the correlations and an asterisk is added for p < 0.010*.

*Also in the present group, copper correlated with ceruloplasmin and peroxides, as found by Altamura et al. study (10), who observed Cu was strictly correlated to CP (r = 0.91, p < 0.001) and to peroxides (r = 0.68, p < 0.001)*.

### Copper and Clinical Recovery

**Figure 1:**
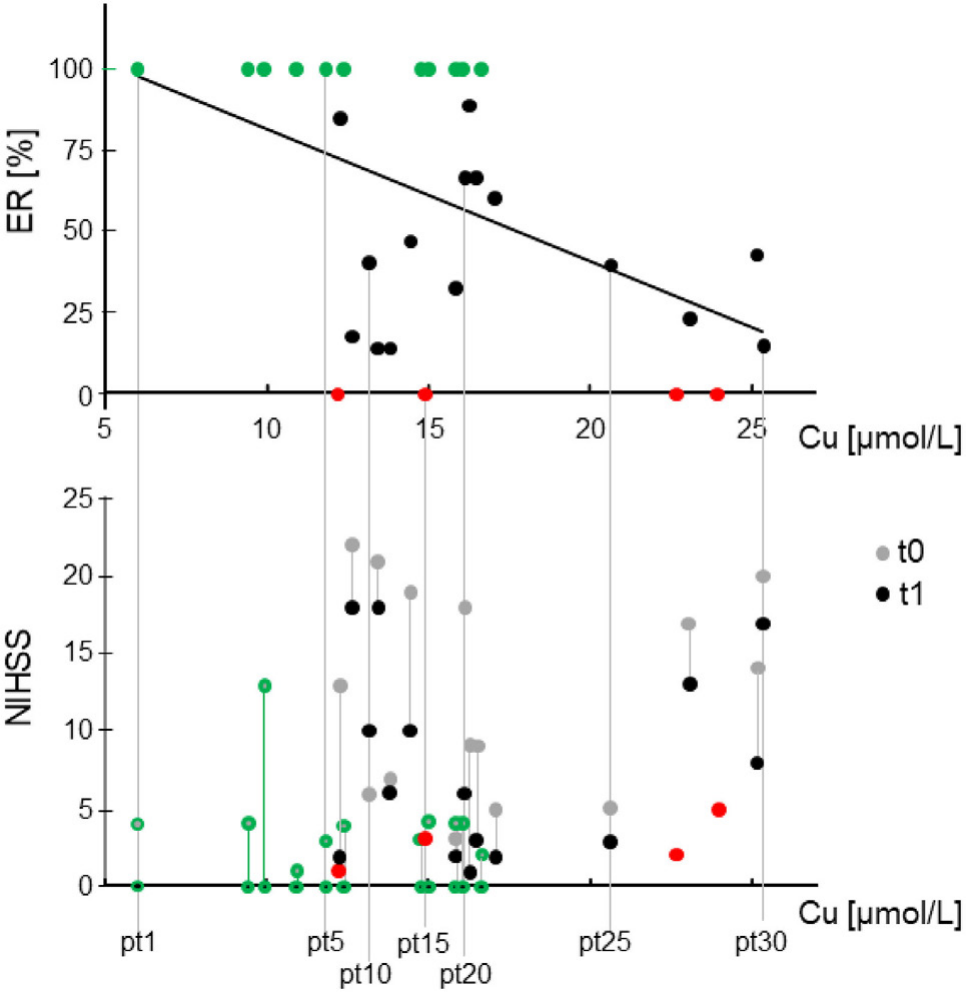
Relationship between stroke acute serum copper and recovery and clinical states. Top: scatter plot of the effective recovery (ER) in relationship with the total copper in acute phase, the only variable entering the regression model explaining 24% of ER variance. Bottom: clinical state scored by National Institute of Health Stroke Scale (IHSS) in acute (t0) and stabilized phase (t1) expressed in function of the total copper (*x*-axis) to identify single patients in terms of recovery (top section) and clinical states (bottom). It is visible that the absence of correlation of total copper with NIHSS at t0. It is also noticeable that people with total copper above 20 µmol/L in t0 recover poorly, in particular patients 26 and 28 (order determined by copper level) had minimal and mild symptoms in t0 which did not ameliorate at all. In red, the four people who did not recover at all, i.e., ER = 0, NIHSS at t0 = NIHSS at t1. In green (dots top, contours bottom), the 11 people who recovered completely (ER = 1, NIHSS at t1 = 0).

Higher recovery outcomes correlated with lower levels of copper in the acute phase, with Pearson’s *r* = −0.492, *p* = 0.006. Copper did not display a significant correlation with NIHSS0 (Table 3). Note that the four people who did not recover at all (i.e., they had equal clinical states in the acute and stabilized phases, ER = 0, red dots in Figure 1), had low clinical disability in the acute phase (NIHSS <5). This is also the case for 10 out of the 11 people who recovered completely (green dot and contour in Figure 1). These two facts underline further that the phenomena leading to acute phase clinical severity are largely uncoupled from those that sustain recovery. Considering the inverse relationship between copper and recovery at the individual level, people reaching ER = 1 always had copper levels below 16.6 µmol/L (Figure 1).

Using a regression model, we also checked whether copper could explain ER outcome. This model had ER as a dependent variable and included copper and NIHSS in t0 as the independent variables. It revealed that copper only entered the model, explaining part of ER variation, as expressed by:

ER=1.22−0.041 copper.

This model explained the 24% of ER variance [*F*(1,28) = 8.922, *p* = 0.006, Figure 1], and indicated that a 10% ER loss in the stabilized phase corresponded to a 2.5 µmol/L increased copper in the acute phase.

## Discussion

The main result of our study is that elevation of serum copper measured in the acute phase provided information about a less successful clinical recovery after ischemic stroke, as assessed through the ER index. More specifically, serum copper entered a regression model explaining 24% of ER variance.

We can conjecture about this result, integrating another result, which is the observation of high nCp-Cu levels in the acute phase. This result is in line with a recent study that investigated in depth the speciation of the circulating copper components in ischemic stroke patients (13). Lai and collaborators reported increased levels of the fraction named “small molecule copper” (SMC—copper bound to small peptides and amino acids, a sub-fraction of nCp-Cu), as revealed through a direct technique for low-molecular weight copper measurements. These authors also found increased levels of serum copper, of copper in urine collected in the 24 h and increases in ceruloplasmin activity. In line with these results, we found increased levels of nCp-Cu in the acute phase. With respect to Lai and colleagues’ study, we extended our period of observation to the stabilized phase and focused on the ER at 6 months from the disease onset. Of note is the completely different trend of copper in the clinical picture at t0 compared to t1. The fact that the metal is unrelated to NIHSS in the acute phase (Figure 1), but is associated with recovery (ER) after 6 months, suggests that copper might enter the plasticity phenomena of remodeling supporting stroke recovery. It is known that brain plasticity is a major determinant of the variability of patients’ recovery with similar initial conditions (23, 24). Within plastic phenomena, excitotoxicity plays a relevant role. It encompasses synaptic failure, mitochondrial dysfunctions, lysosomal dysfunctions, and oxidative stress as well as multiple alterations in channel activity. It is the major determinant of neuronal loss after acute stroke. A recent experimental study showed that rats given 100 mg/kg bwt/day CuSO4 (in water by oral gavage) encountered excitotoxicity mechanisms of apoptosis and neuronal death, tied to glutamate and oxidative stress pathways (7). Body copper, particularly in the brains of the experimental animals, was increased with respect to the controls, whereas *N*-methyl-d-aspartate receptors were decreased. The literature concurs, showing that neuronal synaptic plasticity, long-term potentiation, and synaptic failure, which is a cause of persistent symptoms of stroke patients (23, 24, 37–40), encompass the involvement of copper in glutamate-mediated neurotransmission (7, 15–17). Along the same line, in a previous study, we observed that healthy subjects exhibiting increased levels of body copper reserves, as marked by copper and ceruloplasmin values, had lower cortical glutamatergic responsiveness as revealed by a magnetoencephalographic index representative of glutamate-mediated excitability of the primary somatosensory cortex (19) [for review see Ref. (15)].

Another result of our study regards the association of peroxides, iron, and TAS with clinical severity in the acute phase. Nevertheless, they do not correlate with clinical recovery. Thus, current data confirm our previous findings on the association of oxidative stress and iron compounds with ischemic stroke in the acute phase (10), as discussed in that previous report.

Our study has a number of limitations including the small size of the sample, which calls for confirmation of these results in larger and independent cohorts. Furthermore, the clinical assessment through NIHSS is a suitable scoring method in the acute state, but it only roughly assesses the finer functionality of the patient in the stabilized condition. Since the present investigation focuses on acute phase markers correlated with the improvement of the clinical state, we opted to obtain a relative index of the clinical improvement reached by the patient (normalized by the total possible improvement), instead of using scales more appropriate for assessing the patient’s functional abilities and everyday independence in t1 (Modified Rankin Scale, Barthel Index, Fugl-Meyer). These are not collected in the acute phase, thus they do not allow a differential t1 versus t0 evaluation. Our approach looks at acute prognostic factors, for example, before the move of the patient from the hospitals, where the acute phase is treated to the rehabilitation clinic. The acute phase immediately following a stroke event is a time of extreme dynamic evolution in a patient’s conditions. The first 3 days after symptoms onset is a period in which standard treatments are applied to try to limit lesion dimension and to support healing, while other interventions could interact, impairing the vital parameter stabilization. In the following days (3–7 days), the clinical state of a certain patient could locate that patient on one of several possible recovery paths, thus providing quantitative clues to late prognosis. Our goal is to interact with this post-stroke phase, to exploit more actively the features of the acute conditions, specifically those that have a prognostic value, to build additional early interventions specifically aimed at raising patients’ recovery chances toward higher clinical improvement. This is the reason why we used standard scales in the acute phase. In addition, a fine sampling both for serum markers and clinical scores in the early stages (multiple blood samples in the early days, multiple clinical evaluations along the highly dynamic recovery process) can enhance understanding of the effects of copper involvement in plasticity phenomena.

Our study has the strength of focusing on recovery prognosis and potentially opens the way to new applications of treatments that counteract copper excess to preserve neuronal integrity and sustain rehabilitative processes in an area beset with treatment failures.

## Ethics Statement

The Ethics Committees of our Hospitals approved the experimental protocol and all subjects signed a written informed consent before participating, in accordance with the Declaration of Helsinki.

## Author Contributions

GA and NG: patient enrollment and clinical evaluation. RS, MS, and MR: blood sample collection and biochemical evaluation. RS, FZ, and FT: study design, statistical analysis, data interpretation, and manuscript preparation.

## Conflict of Interest Statement

RS receives no consult fee; equity: she has 3% shares in Canox4drug SPA, but does not receive monetary compensation; 3% shares in IGEA Research Corporation but does not receive monetary compensation. Other authors have no conflict of interest.
